# 3-Methyl-2-(3,3,3-tri­chloro-2-hy­droxy­prop­yl)quinazolin-4(3*H*)-one

**DOI:** 10.1107/S1600536813016073

**Published:** 2013-06-15

**Authors:** Fotima Rabbimovna Utayeva, Rasul Yangiberdievich Okmanov, Nuridin Isomidinovich Mukarramov, Khusnutdin Muhitovich Shakhidoyatov, Bakhodir Tashkhodjaev

**Affiliations:** a S.Yunusov Institute of the Chemistry of Plant Substances, Academy of Sciences of Uzbekistan, Mirzo Ulugbek Str. 77, Tashkent 100170, Uzbekistan

## Abstract

The title mol­ecule, C_12_H_11_Cl_3_N_2_O_2_, contains planar quinazolin-4(3*H*)-one (r.m.s. deviation = 0.0257 Å) and propyl fragments, forming a dihedral angle of 10.4 (2)°. An intra­molecular O—H⋯N hydorgen bond occurs. In the crystal, O—H⋯O hydrogen bonds link the mol­ecules into an infinite chain running parallel to the *b* axis.

## Related literature

For the biological properties of quinazolin-4(3*H*)-one deriv­atives, see: Yang *et al.* (2009 ▶). For the fungicidal and insecticidal activity and syntheses of quinazolin-4(3*H*)-one derivatives, see: Shakhidoyatov (1988 ▶). For related structures of quinazolin-4(3*H*)-one derivatives, see: Tashkhodjaev *et al.* (2001 ▶). For hydrogen-bond motifs, see: Bernstein *et al.* (1995 ▶).
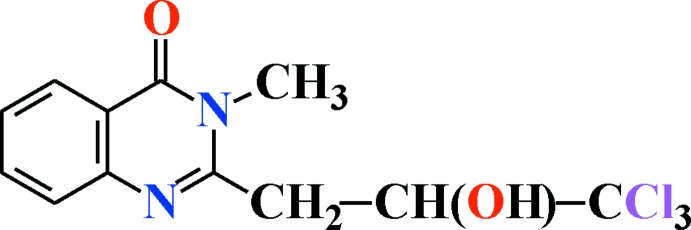


## Experimental

### 

#### Crystal data

C_12_H_11_Cl_3_N_2_O_2_
*M* *_r_* = 321.58
Orthorhombic, 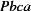

*a* = 9.3440 (19) Å
*b* = 11.352 (2) Å
*c* = 25.719 (5) Å
*V* = 2728.2 (10) Å^3^
*Z* = 8
Cu *K*α radiation
μ = 6.09 mm^−1^
*T* = 291 K
0.23 × 0.20 × 0.18 mm

#### Data collection

Oxford Diffraction Xcalibur Ruby diffractometer
Absorption correction: multi-scan (*CrysAlis PRO*; Oxford Diffraction, 2009 ▶) *T*_min_ = 0.284, *T*_max_ = 0.334
8631 measured reflections
2833 independent reflections
2273 reflections with *I* > 2σ(*I*)
*R*_int_ = 0.032

#### Refinement

*R*[*F* ^2^ > 2σ(*F* ^2^)] = 0.040
*wR*(*F* ^2^) = 0.116
*S* = 1.03
2833 reflections
177 parameters
H atoms treated by a mixture of independent and constrained refinement
Δρ_max_ = 0.34 e Å^−3^
Δρ_min_ = −0.43 e Å^−3^

### 

Data collection: *CrysAlis PRO* (Oxford Diffraction, 2009 ▶); cell refinement: *CrysAlis PRO*; data reduction: *CrysAlis PRO*; program(s) used to solve structure: *SHELXS97* (Sheldrick, 2008 ▶); program(s) used to refine structure: *SHELXL97* (Sheldrick, 2008 ▶); molecular graphics: *XP* in *SHELXTL* (Sheldrick, 2008 ▶); software used to prepare material for publication: *SHELXTL* and *publCIF* (Westrip, 2010 ▶).

## Supplementary Material

Crystal structure: contains datablock(s) I, global. DOI: 10.1107/S1600536813016073/pv2636sup1.cif

Structure factors: contains datablock(s) I. DOI: 10.1107/S1600536813016073/pv2636Isup2.hkl

Supplementary material file. DOI: 10.1107/S1600536813016073/pv2636Isup3.cml

Additional supplementary materials:  crystallographic information; 3D view; checkCIF report

## Figures and Tables

**Table 1:** Hydrogen-bond geometry (Å, °)

*D*—H⋯*A*	*D*—H	H⋯*A*	*D*⋯*A*	*D*—H⋯*A*
O2—H2⋯N1	0.79 (3)	2.47 (3)	2.924 (2)	118 (3)
O2—H2⋯O1^i^	0.79 (3)	2.13 (3)	2.842 (2)	149 (3)

Symmetry code: (i) 
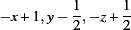
.

**Fig. 1.**
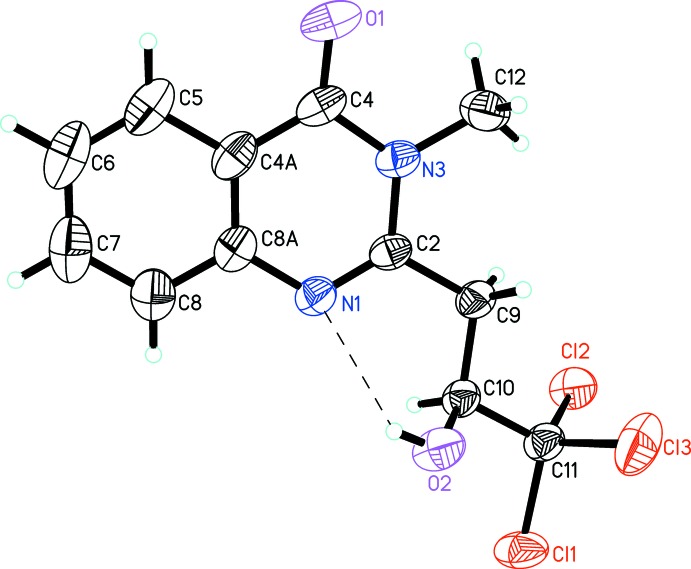
The molecular structure of the title compound, showing the atom numbering scheme. The displacement ellipsoid are drawn at the 50% probability level. Intramolecular H-bond is shown with dashed lines.

**Fig. 2.**
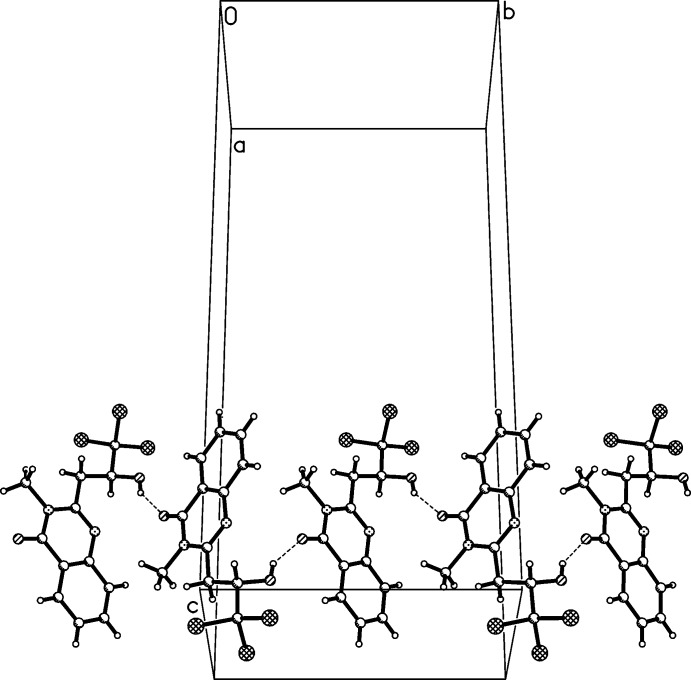
A view of the O—H···O hydrogen bonds (dotted lines) in the crystal structure of the title compound.

**Fig. 3.**
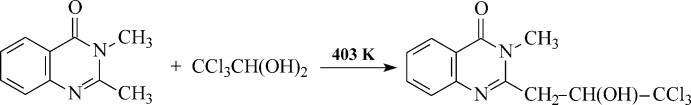
Reaction scheme.

## supplementary crystallographic information

### Comment

Quinazolin-4(3*H*)-one and its derivatives are well known to have broad biological activity, such as antibacterial, antifungal, antimicrobial, anticonvulsant and others (Yang *et al.*, 2009). As well as among quinazolin-4(3*H*)-one derivatives found fungicide, insecticide active compounds for agriculture (Shakhidoyatov, 1988). Reaction of 2,3-dimethylquinazolin-4(3*H*)-one with chloral hydrate in the of reagent–substrate 1:1.2 ratio resulted in the title compound. The structure of the received product was investigated by X-ray diffraction, ^1^H NMR, UF and IR methods.

The bond distances and angles in the title compound (Fig. 1) agree very well with the corresponding bond distances and angles reported in closely related compounds (Tashkhodjaev *et al.*, 2001). The molecule has planar quinazolin-4(3*H*)-one (r.m.s. deviation of 0.0257 Å) and propyl fragment. The angle between planes of quinazolin-4(3*H*)-one ring and *n*-propyl fragment is 10.4 (2)°. The sum of bond angles at atom N3 (close to 360°) and bond length indicate *sp*^2^ hybridization of atom N3. It indicates that the lone electronic pair of nitrogen atom participate in a conjugation with π-electrons of pyrimidine rings.

In the crystal structure of the title compound is observed a cyclic S(6) intramolecular O2—H···N1 hydrogen bond (Bernstein *et al.*, 1995). Another O2—H···O1═C4 hydrogen bond forms an infinite chain along the *b*-axis (Figure 2 & Table 1).

### Experimental

In the pear-shaped flask were placed 2,3-dimethylquinazolin-4(3*H*)-one (0.348 g, 2 mmol) and chloral hydrate (2,2,2-trichloroethane-1,1-diol) (0.397 g, 2.4 mmol) and heated in an oil bath at 403 K for 6 h (Figure 3). The resulting solid was recrystallized from cyclohexane, yield 0.378 g (59%). The purity of synthesized compound was checked by thin layer chromatography (TLC) on plate Whatman AL Sil G/UV (Germany), viewing box 254 nm. Eluents: benzene/acetone (4:1). *R*_f_ 0.65. Colorless crystals, suitable for *X*-ray (in the form of the prisms and with size 0.23x0.20x0.18 mm) were obtained from ethanol at room temperature, m.p. 423–425 K.

### Refinement

The hydrogen atom of the hydroxyl group were located from a difference Fourier synthesis and were allowed to refine. All other H atoms were placed geometrically (with C—H distances of 0.98 Å for CH; 0.97 Å for CH_2_; 0.96 Å for CH_3_; and 0.93 Å for C_ar_) and included in the refinement in a riding motion approximation with *U*_iso_=1.2*U*_eq_(C) [*U*_iso_=1.5*U*_eq_(C,*O*) for methyl H atoms].

### Crystal data

**Table:**

C_12_H_11_Cl_3_N_2_O_2_	*D*_x_ = 1.566 Mg m^−^^3^
*M**_r_* = 321.58	Melting point: 423(2) K
Orthorhombic, *P**b**c**a*	Cu *K*α radiation, λ = 1.54184 Å
Hall symbol: -P 2ac 2ab	Cell parameters from 1044 reflections
*a* = 9.3440 (19) Å	θ = 3.4–35.8°
*b* = 11.352 (2) Å	µ = 6.09 mm^−^^1^
*c* = 25.719 (5) Å	*T* = 291 K
*V* = 2728.2 (10) Å^3^	Prizmatic, colorless
*Z* = 8	0.23 × 0.20 × 0.18 mm
*F*(000) = 1312	

### Data collection

**Table:**

Oxford Diffraction Xcalibur Ruby diffractometer	2833 independent reflections
Radiation source: Enhance (Cu) X-ray Source	2273 reflections with *I* > 2σ(*I*)
Graphite monochromator	*R*_int_ = 0.032
Detector resolution: 10.2576 pixels mm^-1^	θ_max_ = 76.4°, θ_min_ = 3.4°
ω scans	*h* = −10→11
Absorption correction: multi-scan (*CrysAlis PRO*; Oxford Diffraction, 2009)	*k* = −13→14
*T*_min_ = 0.284, *T*_max_ = 0.334	*l* = −32→31
8631 measured reflections	

### Refinement

**Table:**

Refinement on *F*^2^	Primary atom site location: structure-invariant direct methods
Least-squares matrix: full	Secondary atom site location: difference Fourier map
*R*[*F*^2^ > 2σ(*F*^2^)] = 0.040	Hydrogen site location: inferred from neighbouring sites
*wR*(*F*^2^) = 0.116	H atoms treated by a mixture of independent and constrained refinement
*S* = 1.03	*w* = 1/[σ^2^(*F*_o_^2^) + (0.0775*P*)^2^] where *P* = (*F*_o_^2^ + 2*F*_c_^2^)/3
2833 reflections	(Δ/σ)_max_ < 0.001
177 parameters	Δρ_max_ = 0.34 e Å^−^^3^
0 restraints	Δρ_min_ = −0.43 e Å^−^^3^

### Special details

**Table:**

Experimental. ^1^H NMR (400 MHz, DMSO): 8.11 (1*H*, d, J═7.9 Hz, H-5), 7.80 (1*H*, t, J═7.9 Hz, H-7), 7.64 (1*H*, d, J═7.9 Hz, H-8), 7.50 (1*H*, t, J═7.9 Hz, H-6), 7.06 (1*H*, b.d, J═4.8 Hz, OH), 4.84 (1*H*, b.d, J═8.6 Hz, CH-10), 3.46 (1*H*, m, H-9 e), 3.22 (1*H*, dd, J═15.6, J═9.2 Hz, H-9a), 3.6 (3*H*, s, CH_3_-12).In the IR spectrum of the title compound were observed absorption bands due to the stretching vibrations of 3401 sm^-1^ for OH, 2829 sm^-1^ for CH_3_, 1657 sm^-1^ for C═O, 1594 sm^-1^ for C═N, 1563 sm^-1^ for C═C– groups. Additionally, at 695 sm^-1^ was observed p–p valence vibrations of the C—Cl group.The UV spectrum of title compound in ethanol is characterized by absorption bands at 225, 266, 305 and 316 nm.
Geometry. All e.s.d.'s (except the e.s.d. in the dihedral angle between two l.s. planes) are estimated using the full covariance matrix. The cell e.s.d.'s are taken into account individually in the estimation of e.s.d.'s in distances, angles and torsion angles; correlations between e.s.d.'s in cell parameters are only used when they are defined by crystal symmetry. An approximate (isotropic) treatment of cell e.s.d.'s is used for estimating e.s.d.'s involving l.s. planes.
Refinement. Refinement of *F*^2^ against ALL reflections. The weighted *R*-factor *wR* and goodness of fit *S* are based on *F*^2^, conventional *R*-factors *R* are based on *F*, with *F* set to zero for negative *F*^2^. The threshold expression of *F*^2^ > σ(*F*^2^) is used only for calculating *R*-factors(gt) *etc*. and is not relevant to the choice of reflections for refinement. *R*-factors based on *F*^2^ are statistically about twice as large as those based on *F*, and *R*- factors based on ALL data will be even larger.

### Fractional atomic coordinates and isotropic or equivalent isotropic displacement parameters (Å^2^)

**Table:**

	*x*	*y*	*z*	*U*_iso_*/*U*_eq_	
O1	0.5628 (2)	0.70184 (16)	0.27949 (7)	0.0607 (5)	
N1	0.27902 (19)	0.46334 (15)	0.22269 (6)	0.0373 (4)	
C2	0.3539 (2)	0.53163 (17)	0.19344 (7)	0.0331 (4)	
N3	0.44671 (19)	0.61703 (15)	0.21185 (6)	0.0375 (4)	
C4	0.4735 (2)	0.62977 (19)	0.26492 (8)	0.0418 (5)	
C4A	0.3879 (2)	0.5557 (2)	0.29826 (8)	0.0427 (5)	
C5	0.3995 (3)	0.5667 (2)	0.35264 (9)	0.0573 (7)	
H5A	0.4627	0.6208	0.3671	0.069*	
C6	0.3168 (3)	0.4969 (3)	0.38393 (9)	0.0682 (8)	
H6A	0.3253	0.5021	0.4199	0.082*	
C7	0.2197 (3)	0.4179 (3)	0.36200 (10)	0.0640 (8)	
H7A	0.1631	0.3715	0.3836	0.077*	
C8	0.2063 (3)	0.4073 (2)	0.30879 (9)	0.0508 (6)	
H8A	0.1406	0.3548	0.2946	0.061*	
C8A	0.2923 (2)	0.47628 (19)	0.27638 (8)	0.0396 (5)	
C9	0.3415 (2)	0.52114 (18)	0.13538 (7)	0.0379 (4)	
H9A	0.4362	0.5114	0.1206	0.046*	
H9B	0.3010	0.5933	0.1215	0.046*	
C10	0.2482 (2)	0.41768 (17)	0.11937 (7)	0.0345 (4)	
H10A	0.1545	0.4256	0.1361	0.041*	
C11	0.2271 (2)	0.41360 (18)	0.06012 (7)	0.0388 (4)	
C12	0.5242 (3)	0.6964 (2)	0.17710 (9)	0.0499 (6)	
H12A	0.4707	0.7066	0.1455	0.075*	
H12B	0.6163	0.6635	0.1692	0.075*	
H12C	0.5365	0.7714	0.1938	0.075*	
O2	0.3064 (2)	0.30816 (13)	0.13248 (6)	0.0496 (4)	
Cl1	0.10438 (8)	0.30035 (5)	0.04356 (2)	0.0569 (2)	
Cl2	0.15423 (7)	0.54822 (5)	0.03761 (2)	0.05238 (19)	
Cl3	0.38904 (8)	0.38646 (8)	0.02721 (3)	0.0712 (2)	
H2	0.326 (3)	0.299 (3)	0.1623 (13)	0.075 (10)*	

### Atomic displacement parameters (Å^2^)

**Table:**

	*U*^11^	*U*^22^	*U*^33^	*U*^12^	*U*^13^	*U*^23^
O1	0.0610 (10)	0.0636 (11)	0.0576 (10)	−0.0084 (10)	−0.0205 (8)	−0.0186 (8)
N1	0.0407 (9)	0.0369 (9)	0.0343 (8)	0.0009 (8)	−0.0039 (7)	−0.0022 (7)
C2	0.0328 (9)	0.0323 (9)	0.0342 (9)	0.0032 (8)	−0.0072 (7)	−0.0039 (7)
N3	0.0378 (8)	0.0373 (9)	0.0372 (8)	−0.0012 (8)	−0.0083 (7)	−0.0037 (7)
C4	0.0425 (11)	0.0428 (11)	0.0401 (10)	0.0062 (10)	−0.0121 (9)	−0.0116 (8)
C4A	0.0443 (11)	0.0494 (12)	0.0345 (10)	0.0150 (10)	−0.0070 (8)	−0.0056 (8)
C5	0.0608 (15)	0.0744 (17)	0.0365 (11)	0.0162 (13)	−0.0099 (11)	−0.0098 (11)
C6	0.0766 (18)	0.093 (2)	0.0348 (11)	0.0242 (18)	−0.0023 (12)	0.0001 (13)
C7	0.0720 (18)	0.0722 (17)	0.0479 (13)	0.0240 (15)	0.0159 (13)	0.0142 (12)
C8	0.0552 (14)	0.0496 (13)	0.0477 (12)	0.0098 (11)	0.0052 (10)	0.0040 (10)
C8A	0.0423 (11)	0.0411 (10)	0.0355 (9)	0.0117 (9)	−0.0026 (8)	−0.0018 (8)
C9	0.0425 (10)	0.0406 (10)	0.0307 (9)	−0.0041 (9)	−0.0069 (8)	−0.0016 (8)
C10	0.0406 (10)	0.0345 (10)	0.0285 (8)	0.0006 (8)	−0.0059 (8)	0.0008 (7)
C11	0.0468 (11)	0.0369 (10)	0.0326 (9)	0.0005 (9)	−0.0060 (8)	−0.0012 (8)
C12	0.0498 (13)	0.0456 (12)	0.0544 (12)	−0.0112 (11)	−0.0069 (10)	0.0012 (10)
O2	0.0734 (11)	0.0352 (7)	0.0403 (8)	0.0076 (8)	−0.0213 (8)	0.0016 (6)
Cl1	0.0771 (4)	0.0438 (3)	0.0499 (3)	−0.0108 (3)	−0.0273 (3)	−0.0026 (2)
Cl2	0.0730 (4)	0.0420 (3)	0.0421 (3)	0.0013 (3)	−0.0189 (3)	0.0088 (2)
Cl3	0.0643 (4)	0.0993 (6)	0.0499 (3)	0.0099 (4)	0.0118 (3)	−0.0174 (3)

### Geometric parameters (Å, º)

**Table:**

O1—C4	1.227 (3)	C8—C8A	1.398 (3)
N1—C2	1.287 (3)	C8—H8A	0.9300
N1—C8A	1.394 (2)	C9—C10	1.520 (3)
C2—N3	1.384 (3)	C9—H9A	0.9700
C2—C9	1.503 (2)	C9—H9B	0.9700
N3—C4	1.395 (2)	C10—O2	1.398 (2)
N3—C12	1.462 (3)	C10—C11	1.537 (3)
C4—C4A	1.443 (3)	C10—H10A	0.9800
C4A—C8A	1.388 (3)	C11—Cl3	1.761 (2)
C4A—C5	1.408 (3)	C11—Cl2	1.770 (2)
C5—C6	1.369 (4)	C11—Cl1	1.774 (2)
C5—H5A	0.9300	C12—H12A	0.9600
C6—C7	1.395 (4)	C12—H12B	0.9600
C6—H6A	0.9300	C12—H12C	0.9600
C7—C8	1.380 (3)	O2—H2	0.80 (3)
C7—H7A	0.9300		
			
C2—N1—C8A	117.84 (18)	N1—C8A—C8	118.7 (2)
N1—C2—N3	124.22 (17)	C2—C9—C10	112.01 (16)
N1—C2—C9	119.43 (17)	C2—C9—H9A	109.2
N3—C2—C9	116.35 (17)	C10—C9—H9A	109.2
C2—N3—C4	121.31 (18)	C2—C9—H9B	109.2
C2—N3—C12	122.21 (17)	C10—C9—H9B	109.2
C4—N3—C12	116.47 (18)	H9A—C9—H9B	107.9
O1—C4—N3	119.3 (2)	O2—C10—C9	113.49 (17)
O1—C4—C4A	125.7 (2)	O2—C10—C11	105.21 (16)
N3—C4—C4A	114.95 (18)	C9—C10—C11	111.45 (16)
C8A—C4A—C5	120.6 (2)	O2—C10—H10A	108.9
C8A—C4A—C4	119.60 (19)	C9—C10—H10A	108.9
C5—C4A—C4	119.7 (2)	C11—C10—H10A	108.9
C6—C5—C4A	119.3 (3)	C10—C11—Cl3	111.79 (15)
C6—C5—H5A	120.4	C10—C11—Cl2	110.34 (14)
C4A—C5—H5A	120.4	Cl3—C11—Cl2	108.92 (12)
C5—C6—C7	120.1 (2)	C10—C11—Cl1	110.05 (14)
C5—C6—H6A	119.9	Cl3—C11—Cl1	108.24 (11)
C7—C6—H6A	119.9	Cl2—C11—Cl1	107.38 (12)
C8—C7—C6	121.1 (3)	N3—C12—H12A	109.5
C8—C7—H7A	119.5	N3—C12—H12B	109.5
C6—C7—H7A	119.5	H12A—C12—H12B	109.5
C7—C8—C8A	119.4 (3)	N3—C12—H12C	109.5
C7—C8—H8A	120.3	H12A—C12—H12C	109.5
C8A—C8—H8A	120.3	H12B—C12—H12C	109.5
C4A—C8A—N1	121.8 (2)	C10—O2—H2	116 (2)
C4A—C8A—C8	119.5 (2)		
			
C8A—N1—C2—N3	0.0 (3)	C5—C4A—C8A—N1	−179.8 (2)
C8A—N1—C2—C9	−179.45 (18)	C4—C4A—C8A—N1	1.8 (3)
N1—C2—N3—C4	4.6 (3)	C5—C4A—C8A—C8	0.5 (3)
C9—C2—N3—C4	−175.97 (19)	C4—C4A—C8A—C8	−178.0 (2)
N1—C2—N3—C12	−176.9 (2)	C2—N1—C8A—C4A	−3.1 (3)
C9—C2—N3—C12	2.6 (3)	C2—N1—C8A—C8	176.62 (19)
C2—N3—C4—O1	175.7 (2)	C7—C8—C8A—C4A	−1.2 (3)
C12—N3—C4—O1	−2.9 (3)	C7—C8—C8A—N1	179.1 (2)
C2—N3—C4—C4A	−5.5 (3)	N1—C2—C9—C10	−5.9 (3)
C12—N3—C4—C4A	175.87 (18)	N3—C2—C9—C10	174.59 (16)
O1—C4—C4A—C8A	−178.8 (2)	C2—C9—C10—O2	−65.8 (2)
N3—C4—C4A—C8A	2.5 (3)	C2—C9—C10—C11	175.64 (17)
O1—C4—C4A—C5	2.7 (3)	O2—C10—C11—Cl3	−58.26 (19)
N3—C4—C4A—C5	−176.0 (2)	C9—C10—C11—Cl3	65.1 (2)
C8A—C4A—C5—C6	0.9 (4)	O2—C10—C11—Cl2	−179.63 (14)
C4—C4A—C5—C6	179.3 (2)	C9—C10—C11—Cl2	−56.2 (2)
C4A—C5—C6—C7	−1.5 (4)	O2—C10—C11—Cl1	62.0 (2)
C5—C6—C7—C8	0.8 (4)	C9—C10—C11—Cl1	−174.54 (14)
C6—C7—C8—C8A	0.5 (4)		

### Hydrogen-bond geometry (Å, º)

**Table:**

*D*—H···*A*	*D*—H	H···*A*	*D*···*A*	*D*—H···*A*
O2—H2···N1	0.79 (3)	2.47 (3)	2.924 (2)	118 (3)
O2—H2···O1^i^	0.79 (3)	2.13 (3)	2.842 (2)	149 (3)

Symmetry code: (i) −*x*+1, *y*−1/2, −*z*+1/2.
